# Community-level impacts of the coronavirus pandemic on malaria prevention and health-seeking behaviours in rural Benin: A mixed methods study

**DOI:** 10.1371/journal.pgph.0001881

**Published:** 2023-05-19

**Authors:** Claudia Duguay, Manfred Accrombessi, Ludovic K. N’Tcha, Bruno A. Akinro, Edouard Dangbenon, Landry Assongba, Samantha C. Yee, Cindy Feng, Ronald Labonte, Alison Krentel, Natacha Protopopoff, Martin Akogbeto, Manisha A. Kulkarni

**Affiliations:** 1 School of Epidemiology and Public Health, University of Ottawa, Ottawa, Canada; 2 Faculty of Infectious and Tropical Diseases, Disease Control Department, London School of Hygiene and Tropical Medicine, London, United Kingdom; 3 Centre de Recherche Entomologique de Cotonou, Cotonou, Benin; 4 Laboratory of Applied Anthropology and Education for Sustainable Development, University of Abomey-Calavi, Abomey-Calavi, Benin; 5 Department of Community Health & Epidemiology, Dalhousie University, Halifax, Canada; 6 Bruyère Research Institute, Ottawa, Ontario, Canada; Makerere University School of Public Health, UGANDA

## Abstract

Globally, negative impacts of the COVID-19 pandemic on malaria prevention and control efforts have been caused by delayed distributions of long-lasting insecticidal nets (LLIN), decreased outpatient attendance, and disruptions to malaria testing and treatment. Using a mixed methods approach, we aimed to evaluate the impact of COVID-19 on community-level malaria prevention and health-seeking practices in Benin more than one year after the start of the COVID-19 pandemic. We collected data through community-based cross-sectional surveys with 4200 households and ten focus group discussions (FGDs). Mixed effect logistic regression models accounting for a clustered sampling design were used to identify variables associated with main outcomes (good COVID-19 knowledge, LLIN usage and access, and avoidance of health centres). Consistent with the experiences of FGD participants, receiving information from radios or televisions was significantly associated with good COVID-19 knowledge and avoiding health centres because of the pandemic (p<0.001 for both). Qualitative findings also revealed varying and polarizing changes in health-seeking behaviours with participants noting that they either did not change their health-seeking behaviours or went to health centres less or more often because of the pandemic. LLIN usage and access did not decrease in the study area because of the pandemic (LLIN usage: 88% in 2019 to 99.9% in 2021; LLIN access: 62% in 2019 to 73% in 2021). An unexpected change and unintended challenge for sustained malaria prevention included families socially distancing in their homes, resulting in a shortage of LLINs. Our findings showed that there were minimal community-level impacts of the coronavirus pandemic on malaria prevention and health seeking behaviours in rural Benin, which highlights the importance of efforts to sustain malaria prevention and control interventions in the context of the COVID-19 pandemic.

## Background

In 2020, there was an increase in the number of malaria cases and deaths in sub-Saharan African (SSA) countries compared to 2019 –with malaria control having already been stalled since 2015 [1]. In 2020, more than 240 million malaria cases were estimated, with over 600,000 deaths [2]. Benin, along with 29 other countries predominantly in SSA, accounts for 95.7% of malaria deaths globally [2]. Three important tools to control and prevent malaria have been introduced in the past two decades, including long-lasting insecticidal nets (LLIN), early diagnosis with rapid diagnostic tests, and treatment with artemisinin-based combination therapy (ACT) [3]. In Benin, LLINs are a key vector control strategy, with a bed net campaign every three years (most recently in 2020) that is supplemented with targeted LLIN delivery services for children under five and pregnant women [4, 5].

As of April 2020, the coronavirus disease (COVID-19) had spread to all malaria-endemic countries [1]. Benin reported its first COVID-19 case on March 9^th^, 2020, three weeks before its planned LLIN distribution [6]. As of April 26, 2022, Benin had a total of 26,952 recorded cases and 163 recorded deaths due to COVID-19 with three surges of new cases followed by declines: a wave in February 2021, a second wave in August/September 2021, and a third wave in January 2022 [7, 8]. Benin received its first shipment of COVID-19 vaccines in March 2021, and as of May 1, 2022, 23% of the population in Benin were fully vaccinated [4, 5, 9].

Early public health messaging in many malaria-endemic countries during the COVID-19 pandemic, including Benin, was to stay home rather than seek care if someone was experiencing a fever to prevent COVID-19 transmission [1]. However, this message was quickly reversed to continue with suggested malaria control [10]. It is critical to respond to the COVID-19 pandemic, while also continuing to prevent and control malaria [11], yet malaria cases and deaths in the WHO African region both increased between 2019 to 2020 from 213 million to 228 million and 534 000 to 602 000, respectively [2]. This could be attributed to a decrease in outpatient attendance, malaria testing during the initial phase of the pandemic, or disruptions in LLIN distribution [2].

Using a mixed methods approach, we aim to assess the impact of COVID-19 on community-level malaria prevention and health-seeking practices by evaluating factors influencing COVID-19 knowledge, the impact of COVID-19 on LLIN usage and access, and avoidance of healthcare centres because of the pandemic.

## Methods

### Study setting

This study was conducted in three of the nine districts in the Zou department in central Benin: Covè, Zagnanado, and Ouinhi. The Zou department is made up of 850,000 inhabitants with only one major ethnic group (Fon) representing 92.3% of the department [12]. The most cultivated items in the three study districts are cassava (Ouinhi and Zagnanado) and peanuts (Covè) [12]. Malaria transmission is year-round and the incidence per 100 population in the Zou department in 2019 was higher than the national incidence among children one- to four-years old (80.8% compared to 54.2%), and five- to fourteen-years old (24.1% compared to 21.2%), as reported by the 2019 Annuaire des statistiques [13].

**Fig 1:**
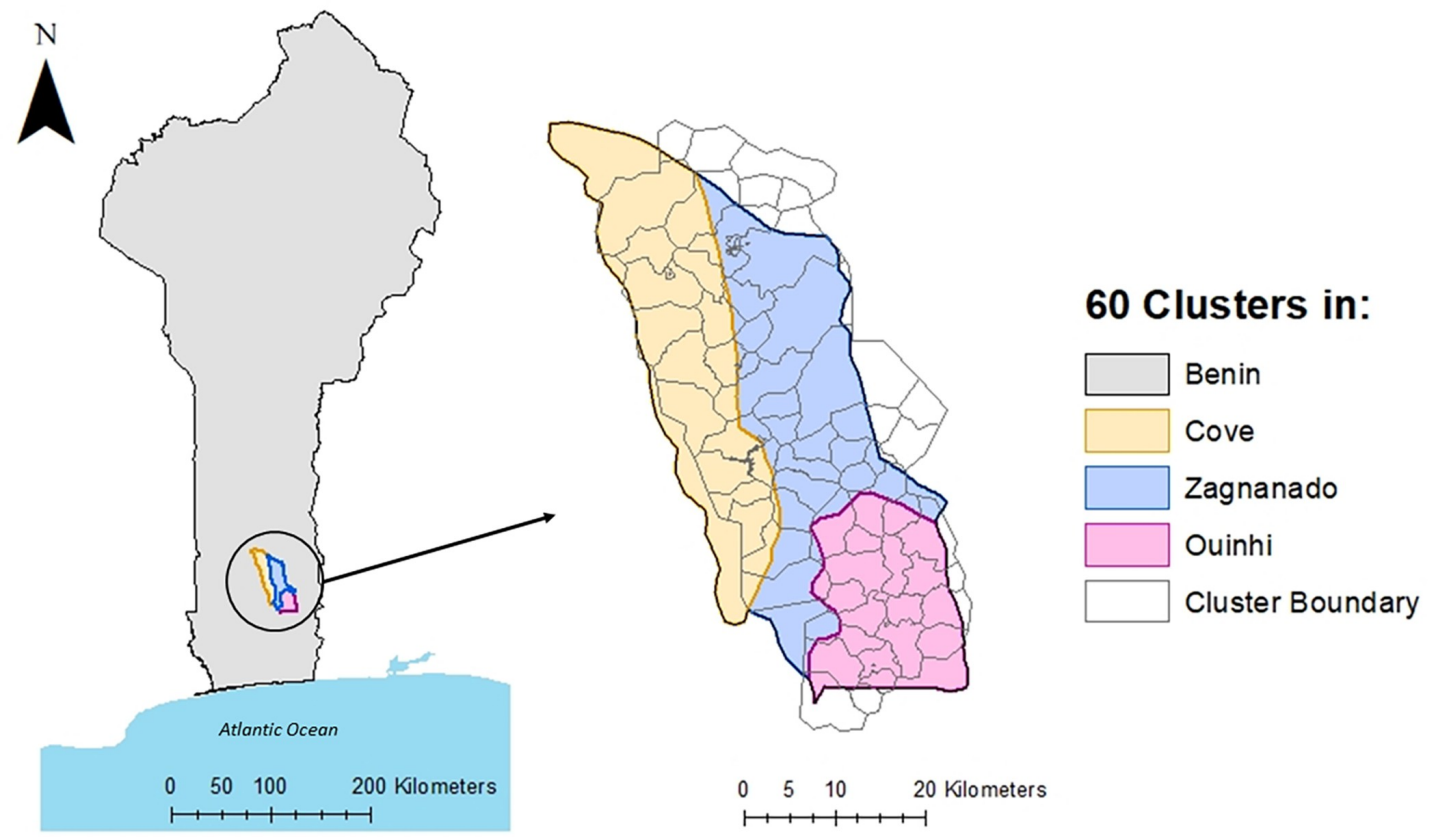
Study area map highlighting Benin and the 60 study clusters within three districts of interest: Zagnanado, Cove, and Ouinhi. Map content was produced with Esri ArcGIS software using study data and data provided by GADM and Natural Earth available online: https://gadm.org/download_country.html and https://www.naturalearthdata.com/.

The study area in the three districts was divided into 60 clusters according to the main study protocol with each cluster comprising one village or group of villages for an average of 200 households (approximately 1,200 residents) (Fig 1) [14].

### Study design

This study was a convergent parallel mixed-methods design that collected quantitative data through a cross-sectional community-based study nested in an ongoing three-arm, single-blinded, parallel, cluster randomized control trial assessing the efficacy of two dual active-ingredients LLINs for the control of malaria and qualitative data through Focus Group Discussions (FGDs) [14]. Data on household demographics, assets, and location, as well as malaria prevention and care-seeking practices were collected through the main trial. For the current study, ten FGDs and supplemental COVID-19 Knowledge, Attitude, Practice (KAP) survey questions were added to the 2021 cohort and 2021 cross-sectional survey instruments.

The surveys were administered by trained field team workers to 2,400 head of households (HoHs) from the cross-sectional surveys and 1,800 HoHs from the cohort follow-up activity. Residents were eligible if they lived in the village during the previous 3 months, provided written consent, and if their children did not have severe illness. The surveys were captured in electronic forms on smartphones installed with Open Data Kit (ODK) collected and stored on a secure server located at the London School of Hygiene and Tropical Medicine (LSHTM).

Ten FGDs of ten people (five groups of ten women and five groups of ten men) were conducted in the three districts in rural Benin (four in Covè, four in Zagnanado, and two in Ouinhi). The FGDs were facilitated by two investigators: the first one moderating the discussion and the second one taking notes. The moderator used a topic guide (in French), and the participants responded and discussed either in French or in their local language. As required, a translator translated the questions from French to the respective local language, then repeated the answers for the moderator from the local language to French. The FGDs were recorded, transcribed verbatim, translated to French, captioned, and validated for the analysis. Select quotes were translated from French to English for their inclusion in this article.

### Qualitative approach

A preliminary codebook for the FGDs was created deductively based on the FGD guidebook and further refined inductively to identify themes and subthemes that arose from reading the transcripts. Two researchers independently coded a selection of two transcripts to ensure interrater reliability. The final codebook was applied to the remaining FGD transcripts using NVivo Qualitative Data Analysis Software (QSR International, Version 12).

### Statistical approach

Data analysis focused on four outcomes: 1) good knowledge of COVID-19, 2) LLIN usage, 3) LLIN access, and 4) avoidance of health centre because of the pandemic. The supplemental COVID-19 KAP survey included twenty-five questions across three domains (symptoms, modes of transmission, and modes of prevention) that had three possible responses: yes, no, and I do not know. We created a score based on the number of correct responses, where a correct response was awarded one point and an incorrect or ‘I don’t know’ response was not awarded any points [15–18]. The total points awarded for each response ranged from 0–25. Using Bloom’s cutoff, good COVID-19 knowledge was defined as a score between 20 and 25, and poor knowledge as a score of 19 or lower [15, 19]. LLIN usage and access reflect important LLIN metrics, given the importance of this intervention as a key malaria prevention and control tool, and are existing indicators defined by the Household Survey Indicators for Malaria Control; LLIN usage is defined as the proportion of individuals who report sleeping under LLINs the previous night, and population LLIN access is defined as the proportion of individuals with access to a LLIN within the households, assuming one LLIN is used by two people [20]. Avoidance of health centres, defined as the proportion of the population who either avoided routine or urgent visits because of the pandemic, was derived from two KAP survey questions (not included in generating good knowledge of COVID-19 score–one addressing avoidance of routine visits (i.e., vaccinations and check-up visits) and another one addressing avoidance of urgent visits (i.e., febrile child)). This metric reflects changes in health-seeking behaviours during the pandemic which points to disruptions in malaria control (i.e., early diagnosis and treatment with ACT).

Candidate explanatory variables hypothesized to be associated with a good knowledge of COVID-19, LLIN usage, LLIN access, and avoidance of health centres include: 1) household demographics (district, ethnicity, marital status, occupation, education), 2) socioeconomic status (SES), 3) population density, 4) distance to nearest health centre facility, 5) source of information of COVID-19 (for good COVID-19 knowledge and avoidance of health centres outcomes only) and 6) good COVID-19 knowledge (avoidance of health centres outcome only). We constructed a wealth score as a proxy for SES using a Principal Components Analysis (PCA) of household assets and dwelling characteristics [21, 22]. The PCA scores were ranked and categorized into quintiles where the first quintile represents the poorest SES group and the fifth quintile the least poor SES group. Data on population density, defined as the number of 100 people per kilometer^2^ in 2020, was retrieved from WorldPop in a grided raster format with a resolution of 1km^2^ [23]. For each household, the population density (as a proxy for urbanicity) was extracted and the Euclidian distance to the nearest health centre (GPS location collected during baseline cross-sectional survey in 2019) was calculated using GIS ArcMap version 10.7.1 (ESRI, Redlands, CA, USA).

Descriptive statistics, including frequencies and proportions for categorical variables and median and interquartile ranges for continuous variables, were generated in SAS version 9.4 (SAS Institute, Cary, NC, USA) (Table 1). Differences between districts and demographic characteristics were compared using a chi-square or a Kruskal Wallis test. P-values less than 0.05 were considered to be statistically significant. Mixed effects logistic regression models accounting for a clustered sampling design were used to identify variables that were associated with each outcome. The “GLMER” function in the lme4 package in RStudio version 4.1.3 (R Core Team, 2018) was used to fit the generalized linear mixed model (GLMM) using the default Laplace approximation, a logit link, and random effects at the cluster level [24]. The univariable association between all candidate explanatory variables and each outcome was assessed and entered into a multivariable model. The “Dredge” function in the MuMIn package in Rstudio version 4.1.3 (R Core Team, 2018) was used to determine the best combination of explanatory variables for each model [25]. The Dredge function is a model selection tool that ranks every candidate model (2^n^ models, where n is the number of predictors in the full model) based on the Akaike Information Criterion (AIC). The model with the lowest AIC was selected and a reduced model was refit using the GLMER function.

**Table 1:** Demographic profile of the study participants by districts (n = 3858).

		Total (n = 3858)	Cove (n = 505)	Ouinhi (n = 1242)	Zagnanado (n = 2111)	P-value*
Ethnicity						
	Fon	2329 (60.4)	355 (70.3)	593 (47.8)	1381 (65.4)	<0.001
	Holli	390 (10.1)	4 (0.8)	269 (21.7)	117 (5.5)	
	Mahi	1083 (28.1)	134(26.5)	369 (29.7)	580 (27.5)	
	Other	56 (1.5)	12(2.4)	11(0.9)	33 (1.6)	
Marital status						
	Married monogamous	2301 (59.6)	322 (63.8)	668 (53.8)	1311 (62.1)	<0.001
	Married polygamous	1120 (29.0)	116 (23.0)	427 (34.4)	577 (27.3)	
	Other	437 (11.3)	67 (13.3)	147 (11.8)	223 (10.6)	
Occupation						
	Farming	2755 (71.4)	327 (64.8)	860 (69.2)	1568 (74.3)	<0.001
	Other	1103 (28.6)	178 (35.3)	382 (30.8)	543 (25.7)	
Education						
	No education	2697 (69.9)	326 (64.6)	846 (68.1)	1525 (72.2)	<0.001
	Some education	1161 (30.1)	179 (35.5)	396 (31.9)	586 (27.8)	
SES						
	Lowest	786 (20.5)	101 (20.2)	212 (17.1)	473 (22.6)	<0.001
	Low	769 (20.1)	69 (13.8)	261 (21.1)	439 (20.9)	
	Average	744 (19.4)	73 (14.6)	280 (22.6)	391 (18.6)	
	High	765 (19.9)	94 (18.8)	375 (22.2)	396 (18.9)	
	Highest	770 (20.1)	162 (32.5)	209 (16.9)	399 (19.0)	
Distance to nearest health facility (km)						
		2.69 (0,14)	1.90 (0,9)	2.50 (0,6)	3.23 (0,14)	<0.001
Population density (per km^2^)						
		238 (29,1181)	417 (42,936)	279 (129,1182)	105 (29,779)	<0.001

Data are displayed as n (%) or median (min, max)

*P-values (significant differences between the three districts, by using the chi squared test for comparison of proportions and Kruskal-Wallis test for medians

**Abbreviations:** SES: socioeconomic status

Model fit was assessed by the area under a receiver operating characteristic curve (AUC) using the GLIMMIX procedure (GLMM using the Laplace approximation) in SAS version 9.4 (SAS Institute, Cary, NC, USA). Spatial autocorrelation was assessed by mapping the regression residuals to identify significant clusters using Global Moran’s I in ArcMap version 10.7.1 (ESRI, Redlands, CA, USA) [26].

### Cluster analysis

Spatial clusters of 1) good COVID-19 knowledge, and 2) avoidance of health centres because of the pandemic were assessed using the Getis-Ord-Gi* in ArcMap version 10.7.1 (ESRI, Redlands, CA, USA) [27]. Getis-Ord-Gi* is a tool that detects statistically significant spatial clusters, which are features of high values surrounded by high values (hotspots) and low values surrounded by low values (coldspots). The output includes z-scores and p-values which indicate either high or low clusters spatially–a p-value less than 0.05 was considered statistically significant.

Data were further analyzed using SaTScan software (v 9.4.1 Kulldorf and Information Management Services, Inc.). A Bernoulli-based probability model using a purely spatial scan was used to identify clusters of high and low rates (i.e., households of good COVID-19 knowledge surrounded by households of good COVID-19 knowledge and households of poor COVID-19 knowledge surrounded by households of poor COVID-19 knowledge). Circular windows of varying radii compared events within the circular window to those expected, with the null hypothesis that there is no difference in the number of events inside and outside the circular window. A relative risk was then generated for each cluster, where a risk ratio (RR) of greater than 1 represents a cluster where the probability of being an event within the circular window is greater than the probability of being an event outside the circular window (RR>1). Results from the Getis-Ord-Gi* and SatScan were then visually inspected to see in what ways they converge or diverge from each other.

### Mixed-methods approach

The themes and subthemes that were identified from the FGDs were then compared with the results from the four statistical models to have a complete understanding of the factors influencing the outcome in each model: 1) good knowledge of COVID-19, 2) LLIN usage, 3) LLIN access, and 4) avoidance of health centre because of the pandemic. We then assessed in what way do the qualitative and quantitative results 1) converge or diverge from each other, 2) relate to one another, and 3) combine to create a better understanding of the results [28].

### Ethics statement

The protocol for this study was reviewed and approved by: the institutional review board of the University of Ottawa (Certificate H-07-20-5944) (Canada); the institutional review board of the London School of Hygiene and Tropical Medicine (LSHTM Ethics Ref: 22637) (United Kingdom); and the ethical review committee of the Benin National Ethics Committee for Health Research (N° 047/MS/DRFMT/CNERS/SA) (Benin). All participants in this study were adults aged 18 years or older. Each participant was read an informed consent form by data collectors, which was read and signed by each participant prior to commencing data collection and focus group discussion.

## Results

### Characteristics of the study population

The majority of HoHs were from the Fon ethnic group (n = 2329, 60%), were in a monogamous marriage (n = 2301, 60%), were farmers (n = 2755, 71%), and had no education (n = 2697, 70%) (Table 1). Households were located at a median Euclidean distance of 2.7 km from a health centre with a median population density of 238 people per km^2^. There was a statistically significant difference between the demographic profiles and all three districts. Notably, the Holli ethnic group was the most represented in Ouinhi (n = 269, 22%), compared to Covè (n = 4, 1%) and Zagnanado (n = 117, 6%). Variability was also present in education across the district with 72% (n = 1525) of HoHs in Zagnanado having no education compared to 68% (n = 846) in Ouinhi and 65% (n = 326) in Covè.

### Factors influencing COVID-19 knowledge

Overall, 9.8% (n = 378) participants had a good knowledge of COVID-19 based on questions surrounding knowledge of symptoms, modes of transmission, and preventative measures of the disease (Table 2). While the minority of HoHs had a good knowledge of COVID-19, the majority were able to identify fevers (n = 2818, 73%) and coughs (n = 2973, 77%) as symptoms of COVID-19, and identified handwashing (n = 3549, 92%) and facemasks (n = 3192, 83%) as methods of prevention of COVID-19 (S1 Table). Key themes from the FGDs surrounding COVID-19 knowledge were the symptoms, modes of transmission, and preventative measures of COVID-19 which provided more context to the quantitative results. Participants frequently mentioned more than one symptom of COVID-19—most often noting fever and coughs, followed by fatigue and headaches. Other notable symptoms of COVID-19 were anemia, sneezing, loss of taste, and vomiting. Participants also noted that COVID-19 was dangerous, destructive, and deadly, however only men noted themes surrounding the financial burden of the disease.

**Table 2:** Emerging themes surrounding COVID-19 knowledge.

	n (%)	Supporting Quote
Good COVID-19 Knowledge	378 (9.8%)	“When someone is infected, and rubs up against me, or even shakes my hand, they can transmit the disease.” Male, Ouinhi
		“The rules like staying 1-meter apart while we travel, mask-wearing, not shaking hands, and not coughing in the air. If we respect these rules, we will stay away from the disease.” Male, Zagnanado
Poor COVID-19 Knowledge	3480 (90.2%)	“What I know from this disease is that when you cough and spit on the ground, and someone sweeps the dust with the spit, this person can inhale the dust and can contract the disease.” Female, Covè
		“We can transmit this disease by our sweat, that is why we need to respect a distance of 1 meter.” Female, Covè

Although participants had a varying understanding of the modes of transmission for COVID-19, they often noted effective preventative measures. The modes of transmission given by participants ranged from direct contact (i.e., handshake, sweat, bumping into an infected person), indirect contact (i.e., dust, an infected persons breath, sneezing or coughing in the air, being near an infected person), and contaminated foods (i.e., bushmeat).

*“If I sneeze or cough in the air, the wind can transport this virus to contaminate others” **Male, Covè***

Despite a poor understanding of the modes of transmission for COVID-19, all participants mentioned “gestes barrières", which include handwashing, mask-wearing, and social distancing, as measures to reduce the spread of COVID-19. Many participants also commented that they installed a handwashing station in their homes to prevent COVID-19, changed the way that they greeted others (i.e., with their elbows or feet), and sneezed and coughed in their masks. Unanticipated preventative measures perceived to be associated with COVID-19 by respondents emerged from the FGDs and included themes surrounding cleanliness (i.e., sweeping around the house, and doing dishes, laundry, and bathing).

*“We organized a group of women to clean up the roads in our village every day” **Male, Covè***

Participants cited radios (specifically spoken in Fon) and public criers (crieur publique) as the most reliable sources of information for disseminating information surrounding COVID-19.

*“According to myself, if the public criers pass the information, a lot of people will be informed and will have the information on time, because not everyone has the money for a radio” **Male, Zagnanado***

To quantitatively understand factors associated with good COVID-19 knowledge, HoH ethnicity, HoH education, distance to the nearest health facility, population density, and select sources of information about COVID-19 (radio, television, health centres, and religious leaders) were included in a multivariable model. HoH education [some vs none] (adjusted odds ratio [aOR] 1.44, 95% CI 1.11–1.86, p<0.001); distance to nearest health facility [per km] (aOR 1.11, 95% CI 1.01–1.23, p = 0.033); radios (aOR 2.23, 95% CI 1.48–3.38, p<0.001), televisions (aOR 2.92, 95% CI 2.18–3.91, p<0.001) and religious leaders (aOR 1.74, 95% CI 1.34–2.27, p<0.001) as sources of information for COVID-19 were significantly associated with a good COVID-19 knowledge (Table 3). Although included in the final multivariable model, ethnicity, and health centres as a source of information were not significantly associated with good COVID-19 knowledge. The final multivariable model had excellent discrimination (AUC = 0.875) and there was no evidence of spatial autocorrelation (p = 0.085).

**Table 3:** Results of mixed effects logistic regression analysis of factors associated with knowledge of the novel coronavirus disease 2019 (n = 3858).

			Univariable Model			Adjusted Model		
		%	OR	(95% CI)	P-value	AOR*	(95% CI)	P-value
District								
	Cove	12	REF	-	-	-	-	-
	Ouinhi	10	0.45	(0.17–1.19)	0.110	-	-	-
	Zagnanado	9	0.64	(0.27–1.54)	0.320	-	-	-
Ethnicity								
	Fon	12	REF	-	-	REF	-	-
	Mahi	6	0.37	(0.26–0.52)	<0.001	0.79	(0.54–1.16)	0.235
	Holli	6	0.44	(0.23–0.78)	<0.001	0.60	(0.33–1.11)	0.105
	Other	7	0.65	(0.19–1.71)	0.419	0.78	(0.26–2.38)	0.665
Marital status								
	Married monogamous	10	REF	-	-	-	-	-
	Married polygamous	10	1.02	(0.80–1.31)	0.862	-	-	-
	Other	6	0.57	(0.37–0.86)	<0.001	-	-	-
Occupation								
	Farming	9	REF	-	-	-	-	-
	Other	11	1.21	(0.93–1.57)	0.150	-	-	-
Education								
	No education	8	REF	-	-	REF	-	-
	Some education	14	1.93	(1.52–2.45)	<0.001	1.44	(1.11–1.86)	<0.001
SES								
	Lowest SES	6	REF	-	-	-	-	-
	Low SES	10	1.49	(1.00–2.22)	0.043	-	-	-
	Average	11	1.74	(1.17–2.61)	<0.001	-	-	-
	High SES	9	1.60	(1.06–2.41)	0.025	-	-	-
	Highest SES	13	2.80	(1.87–4.26)	<0.001	-	-	-
Distance to nearest health facility (km)								
		2.78	1.09	(0.99–1.20)	0.067	1.11	(1.01–1.23)	0.033
Population density (100 people/km^2^)								
		2.66	1.07	(0.97–1.19)	0.160	1.12	(1.00–1.26)	0.053
Source of information								
	Radio							
	No	4	REF	-	-	REF	-	-
	Yes	11	2.96	(2.00–4.51)	<0.001	2.23	(1.48–3.38)	<0.001
	Tv							
	No	8	REF	-	-	REF	-	-
	Yes	25	4.24	(3.19–5.62)	<0.001	2.92	(2.18–3.91)	<0.001
	Health centre							
	No	8	REF	-	-	REF	-	-
	Yes	16	2.50	(1.94–3.22)	<0.001	1.22	(0.92–1.61)	0.161
	Village leaders							
	No	9	REF	-	-	-	-	-
	Yes	12	1.46	(1.12–1.89)	0.004	-	-	-
	Religious leaders							
	No	7	REF	-	-	REF	-	-
	Yes	12	2.06	(1.61–2.65)	<0.001	1.74	(1.34–2.27)	<0.001
	Word of Mouth							
	No	9	REF	-	-	-	-	-
	Yes	10	1.07	(0.79–1.45)	0.670	-	-	-
	Other							
	No	9	REF	-	-	-	-	-
	Yes	25	4.33	(3.11–6.01)	<0.001	-	-	-

*Full models adjusted for intervention arm, survey number, and cluster number

**Abbreviations:** SES: Socioeconomic status, AOR: Adjusted Odds Ratio, OR: Odds Ratio

**Fig 2:**
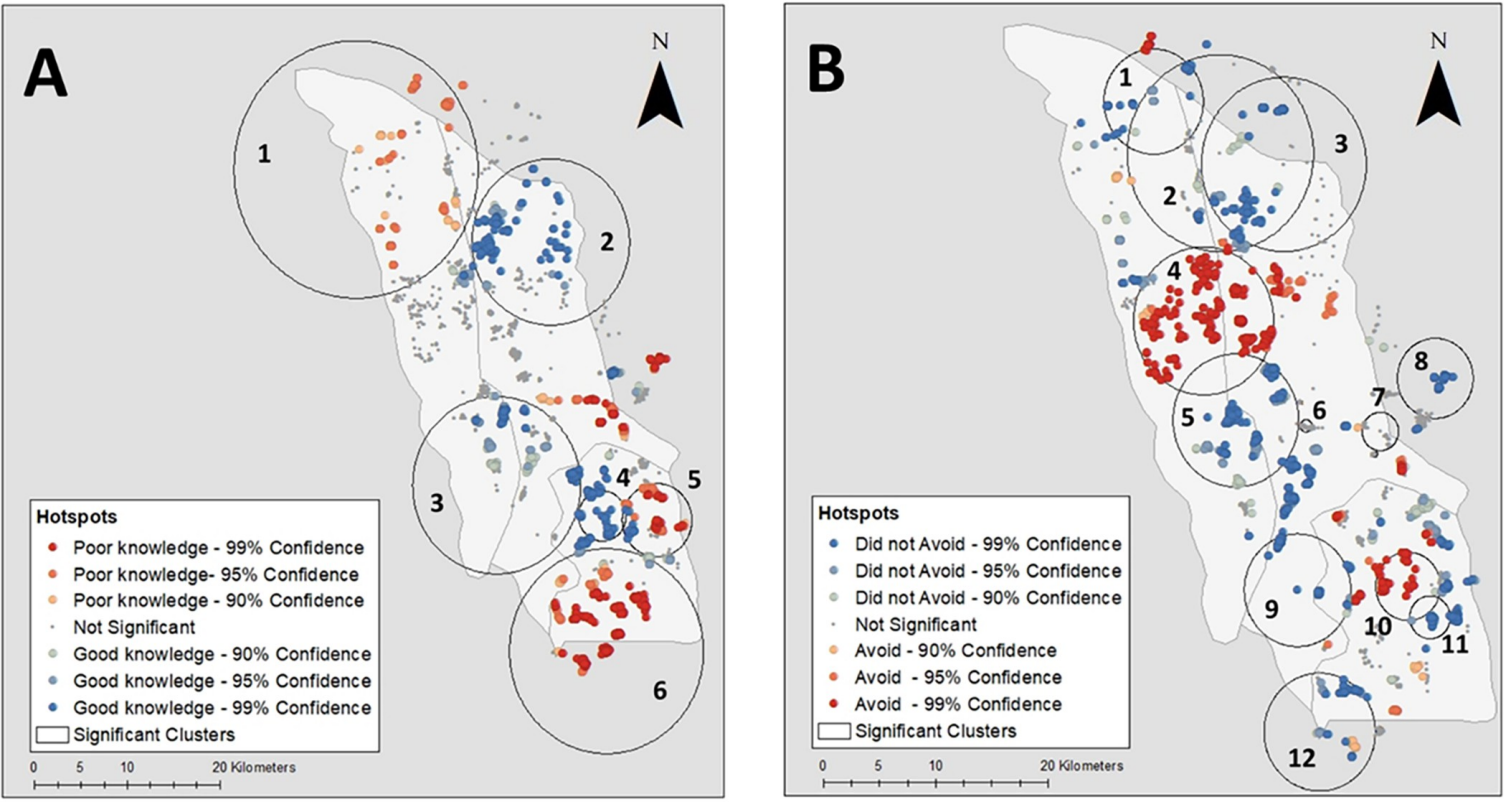
Spatial clusters of good COVID-19 knowledge (A) and avoidance of health centres during the COVID-19 pandemic (B). Map content was produced with Esri ArcGIS software using study data and data provided by GADM available online: https://gadm.org/download_country.html.

The results of the Getis-Ord-Gi* test showed hotspots of households with good and poor COVID-19 knowledge which were overlapped by the locations of statistically significant clusters detected using Kulldorf spatial scan statistic in SaTScan (Fig 2A). The two tests detected similar patterns with significant clusters of good COVID-19 knowledge in Zagnanado North (Cluster 2: RR = 2.38) and Ouinhi North (Cluster 4: RR = 4.67), and significant clusters of poor knowledge in Covè North (Cluster 1: RR = 0.29), and Ouinhi South (Cluster 5: RR = 0 and Cluster 6: RR = 0.03).

### Impact of COVID-19 on LLIN usage and access

**Fig 3:**
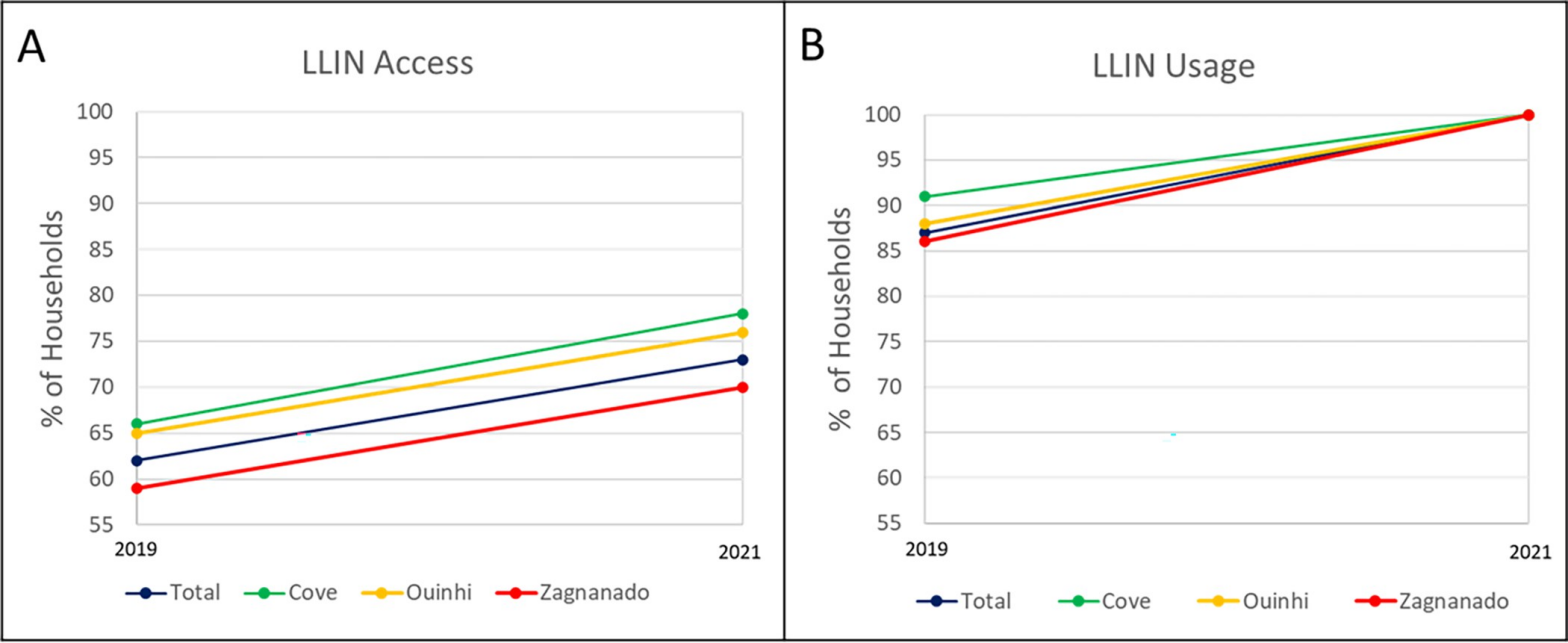
Temporal trends of LLIN access (A) and LLIN usage (B) before and during the COVID-19 pandemic.

LLIN usage and access did not decrease as a result of the pandemic (LLIN usage: 88% in 2019 to 99.9% in 2021; LLIN access: 62% in 2019 to 73% in 2021); indeed, a slight increase in both indices was noted, which likely reflects the LLIN distribution that took place as part of the malaria intervention trial (Table 4). Although not statistically significant, there were differences in LLIN usage and access between the three districts with Covè having greater LLIN usage and access compared to Zagnanado and Ouinhi (Fig 3A and 3B).

**Table 4:** Emerging themes surrounding LLIN usage and access.

	n (%)	Supporting Quote
LLIN Usage	2414 (87.5%) in 2019	“If the child does not sleep under the bed-net, they can have a fever, so have malaria and miss blood.” Female, Covè
	2126 (99.9%) in 2021	
		“If you’re not in the habit of sleeping under an insecticide treated bed-net, the mosquito will bite you.” Male, Covè
No LLIN Usage	346 (12.5%) in 2019	“At nightfall, the kids go play or watch television. In that brief time, they get mosquito bites which brings malaria.” Male, Zagnanado
	3 (0.01%) in 2021	
		“Staying under a bed-net is too warm. We have to stand outside of mosquito nets to get some air.” Male, Covè
LLIN Access	1704 (61.7%) in 2019	No supporting quotes available.
	1550 (72.8%) in 2021	
No LLIN Access	1056 (38.3%) in 2019	“When the coronavirus came, we separated the kids and adults’ beds, we don’t sleep with the kids anymore and they feel better about that.” Female, Covè
	579 (27.2%) in 2021	
		“They said that we should not gather, so we don’t have enough of bed-nets.” Female, Zagnanado
		“My children often have malaria because we don’t have any bed-nets at home.” Female, Zagnanado

At least one participant in every FGD stated that mosquitoes transmit malaria and noted bed nets and other measures to prevent mosquito bites (i.e., weeding, eliminating standing water) as measures to prevent malaria. Malaria was most often noted as the most important disease (i.e., top priority, most prevalent, most deadly), and therefore participants commented on the importance of preventing malaria.

*“To put it simply, malaria is the chief disease in Benin” **Male, Ouinhi***

The barrier that was most often noted in preventing malaria (before the pandemic) related to difficulties in entering a bed net prior to when mosquitoes were active at night. An unexpected change and barrier in malaria prevention during the COVID-19 pandemic was that families were socially distancing in their homes–specifically no longer sharing beds and bed nets, resulting in a shortage of bed nets.

*“We don’t have enough bed-nets because someone that has 10 children and we give them 5 bed-nets and we sleep one child per bed-net, this person is missing 5 bed-nets, as before we only needed 6 to 7 bed-nets. This means that we need to gather the children under one bed-net. So, the risk of getting a mosquito bite is increased.” **Male, Zagnanado***

Since 99.9% of the households in 2021 used a LLIN the night before (LLIN usage), a multivariable model was not run to assess the effect of the pandemic on LLIN usage. However, based on the univariable model, ethnicity [Holli vs Fon] (OR 2.85, 95% CI 1.90–4.31, p<0.001), highest vs lowest SES (OR 2.03, 95% CI 1.40–2.94, p<0.001), high vs lowest SES (OR 2.20, 95% CI 1.53–3.18, p<0.001), average vs lowest SES (OR 1.89, 95% CI 1.32–2.66, p<0.001), and low vs lowest SES (OR 1.58, 95% CI 1.14–2.17, <0.001) were significantly associated with LLIN usage (Table 5).

**Table 5:** Results of mixed effect logistic regression analysis of demographic factors associated with long lasting insecticidal net usage (n = 4889).

			Univariable Model		
		%	OR	(95% CI)	P-value
Time points					
	Pre COVID-19	88	REF	-	-
	Post COVID-19	99.9	109.3	(41–443)	<0.001
District					
	Cove	95	REF	-	-
	Ouinhi	93	0.69	(0.30–1.50)	0.340
	Zagnanado	92	0.65	(0.30–1.40)	0.250
Ethnicity					
	Fon	92	REF	-	-
	Holli	97	2.85	(1.90–4.31)	<0.001
	Mahi	92	0.97	(0.60–1.60)	0.890
	Other	79	0.32	(0.20–0.52)	<0.001
Marital status					
	Married monogamous	94	REF	-	-
	Married polygamous	92	0.82	(0.63–1.08)	0.144
	Other	91	0.66	(0.49–0.91)	<0.001
Occupation					
	Farming	93	REF	-	-
	Other	92	0.89	(0.68–1.20)	0.360
Education					
	No education	92	REF	-	-
	Some education	94	1.30	(0.98–1.70)	0.068
SES					
	Lowest SES	89	REF	-	-
	Low SES	92	1.58	(1.14–2.17)	<0.001
	Average	94	1.89	(1.34–2.66)	<0.001
	High SES	95	2.20	(1.53–3.18)	<0.001
	Highest SES	95	2.03	(1.40–2.94)	<0.001
Population density (100 people/km^2^)					
		2.37	0.97	(0.88–1.10)	0.57

**Abbreviations:** SES: Socioeconomic status, OR: Odds Ratio

To quantitatively investigate the effect of the pandemic on LLIN access, the time point (2021 or 2019), HoH ethnicity, HoH marital status, HoH education, and population density were included in the multivariable model. The time point [2021 (post COVID-19) vs 2019 (pre COVID-19)] (aOR 1.48, 95% CI 1.28–1.70, p<0.001), ethnicity [Holli vs Fon] (aOR 1.44, 95% CI 1.19–1.74, p<0.001), marital status [polygamous vs monogamous] (aOR 0.80, 95% CI 0.69–0.92, p<0.001), marital status [other vs monogamous] (aOR 2.19, 95% CI 1.76–2.73, p<0.001), and population density [per100 people/km^2^](aOR 1.07, 95% CI 1.01–1.12, p = 0.014) were statistically associated with LLIN access (Table 6). Although included in the final multivariable, ethnicity (Mahi vs Fon, and other vs Fon), and HOH education were not significantly associated with LLIN access. The final multivariable model had poor discrimination (AUC = 0.679) and there was no evidence of spatial autocorrelation (p = 0.306).

**Table 6:** Results of mixed effect logistic regression analysis of demographic factors associated with long lasting insecticidal net access (n = 4889).

			Univariable Model			Adjusted Model		
		%	OR	(95% CI)	P-value	AOR*	(95% CI)	P-value
Time points								
	Pre COVID-19	62	REF	-	-	-	-	-
	Post COVID-19	73	1.70	(1.50–1.90)	<0.001	1.48	(1.28–1.70)	<0.001
District								
	Cove	71	REF	-	-	-	-	-
	Ouinhi	70	0.96	(0.62–1.50)	0.860	-	-	-
	Zagnanado	64	0.74	(0.49–1.10)	0.150	-	-	-
Ethnicity								
	Fon	62	REF	-	-	REF	-	-
	Holli	75	1.80	(1.52–2.10)	<0.001	1.44	(1.19–1.74)	<0.001
	Mahi	71	1.20	(0.87–1.60)	0.300	1.09	(0.80–1.48)	0.603
	Other	70	1.10	(0.74–1.60)	0.710	1.24	(0.85–1.80)	0.272
Marital status								
	Married monogamous	66	REF	-	-	REF	-	-
	Married polygamous	59	0.78	(0.68–0.90)	<0.001	0.80	(0.69–0.92)	<0.001
	Other	82	2.22	(1.79–2.80)	<0.001	2.19	(1.76–2.73)	<0.001
Occupation								
	Farming	66	REF	-	-	-	-	-
	Other	68	0.91	(0.79–1.10)	0.220	-	-	-
Education								
	No education	67	REF	-	-	REF	-	-
	Some education	65	0.81	(0.70–0.94)	<0.001	0.89	(0.76–1.03)	0.109
SES								
	Lowest SES	70	REF	-	-	-	-	-
	Low SES	62	0.75	(0.61–0.91)	<0.001	-	-	-
	Average	66	0.83	(0.68–1.02)	0.071	-	-	-
	High SES	66	0.77	(0.62–0.94)	0.011	-	-	-
	Highest SES	70	0.86	(0.69–1.07)	0.172	-	-	-
Population density (100 people/km^2^)								
		2.46	1.10	(1.00–1.10)	<0.001	1.07	(1.01–1.12)	0.014

*Full models adjusted for intervention arm and cluster number

**Abbreviations:** SES: Socioeconomic status, AOR: Adjusted Odds Ratio, OR: Odds Ratio

### Health care avoidance during pandemic

There were varying, and polarizing, changes in health-seeking behaviours because of the pandemic. A fifth of the participants (n = 714, 18.5%) avoided health centres because of the coronavirus 2019 pandemic, and FGD participants noted that they either did not change their health-seeking behaviours, or went to health centres less or more often because of the pandemic (Table 7). Reasons for avoiding health centres because of the pandemic for FDG participants included fear of disease exposure and fear that they would be forced into a quarantine facility. For similar reasons, participants commented that their fear of COVID-19 (i.e., overlapping symptoms, deadly) led them to go to health centres more often because of the pandemic.

**Table 7:** Emerging themes surrounding avoidance of health-centres because of the coronavirus 2019 pandemic.

	n (%)	Supporting Quote
Avoided health-centre	714 (18.5%)	“When your child is sick, they said that we have to go to the hospital, but the child refuses because they said that the health centres is where people can catch the disease [coronavirus].” Female, Zagnanado
		“We don’t have money and when we go to the hospital and you don’t have money, the doctors won’t take care of you.” Female, Zagnanado
Did not avoid health-centre	3144 (81.5%)	“If you have malaria and you don’t go quickly to the hospital, or if it’s a child and you don’t take care of them by taking them to the hospital, you can lose the child.” Female, Covè
		“We used to keep our kids at home…but since the coronavirus came…we go quickly to the hospital to get the child tested for malaria or coronavirus.” Male, Covè

Even before the pandemic, there were polarizing health-seeking behaviours amongst the FGD participants—noting that they either avoid health centres (e.g., too expensive) or go quickly to the health centre when someone is sick (e.g., fear of death, when someone is febrile, to receive treatment).

*“We go to the hospital to treat the children despite the situation with coronavirus” **Female, Ouinhi***

*“What concerns us about malaria is that when you get it, you spend all your money at the hospital, when this happens you have nothing but loss of life, this is what concerns me the most about malaria” **Female, Covè***

To quantitatively understand factors associated with avoidance of health centres, HoH ethnicity, HoH education, SES, good COVID-19 knowledge, and select sources of information about COVID-19 (radio, television, health centres, village leaders, religious leaders, and word of mouth) were included in the multivariable model. Good COVID-19 knowledge (aOR 2.16, 95% CI 1.54–3.04, p<0.001); and radios (aOR 3.04, 95% CI 2.20–4.21, p<0.001) and television (aOR 1.68, 95% CI 1.20–2.33, p<0.001) as sources of information for COVID-19 were significantly associated with avoiding health centres because of the pandemic (Table 8). In contrast, HoH ethnicity [Holli vs Fon] (aOR 0.42, 95% CI 0.31–0.59, p<0.001), HoH education [some vs none] (aOR 0.75, 95% CI 0.58–0.96, p = 0.021), high vs lowest SES (aOR 0.57, 95% CI 0.40–0.81, p<0.001), highest vs lowest SES (aOR 0.67, 95% CI 0.46–0.97, p = 0.037), religious leaders as a source of information for COVID-19 (aOR 0.78, 95% CI 0.61–0.99, p = 0.040) were significantly associated with not avoiding health centres because of the pandemic (Table 8). Although included in the final multivariable, ethnicity (Mahi vs Fon, and other vs Fon, average vs lowest SES, low vs lowest SES, and select sources of information for COVID-19 (health centres, village leaders, and word of mouth) were not significantly associated with avoiding health centres because of the pandemic. The final multivariable model had excellent discrimination (AUC = 0.875) and there was no evidence of spatial autocorrelation (p = 0.957).

**Table 8:** Results of mixed effect logistic regression analysis of factors associated with avoidance of health-centres because of the coronavirus 2019 pandemic (n = 3858).

			Univariable Model			Adjusted Model		
		%	OR	(95% CI)	P-value	AOR*	(95% CI)	P-value
District								
	Cove	17	REF	-	-	-	-	-
	Ouinhi	19	1.20	(0.30–5.31)	0.768	-	-	-
	Zagnanado	19	1.10	(0.32–4.41)	0.831	-	-	-
Ethnicity								
	Fon	19	REF	-	-	REF	-	-
	Mahi	17	1.23	(0.94–1.59)	0.070	0.58	(0.33–1.01)	0.056
	Holli	20	0.62	(0.36–1.03)	0.120	0.42	(0.31–0.59)	<0.001
	Other	18	1.71	(0.74–3.69)	0.180	1.32	(0.56–3.15)	0.524
Marital status								
	Married monogamous	19	REF	-	-	-	-	-
	Married polygamous	18	0.89	(0.72–1.11)	0.400	-	-	-
	Other	19	1.14	(0.84–1.54)	0.300	-	-	-
Occupation								
	Farming	20	REF	-	-	-	-	-
	Other	16	0.95	(0.75–1.20)	0.680	-	-	-
Education								
	No education	20	REF	-	-	REF	-	-
	Some education	15	0.75	(0.60–0.94)	0.010	0.75	(0.58–0.96)	0.021
SES								
	Lowest SES	26	REF	-	-	REF	-	-
	Low SES	22	0.99	(0.75–1.31)	0.947	0.98	(0.72–1.32)	0.922
	Average	18	093	(0.69–1.26)	0.663	0.88	(0.64–1.21)	0.444
	High SES	12	0.61	(0.44–0.85)	<0.001	0.57	(0.40–0.81)	<0.001
	Highest SES	16	1.02	(0.73–1.42)	0.906	0.67	(0.46–0.97)	0.037
COVID-19 Knowledge								
	Poor	18	REF	-	-	REF	-	-
	Good	25	1.47	(1.09–1.99)	0.011	2.16	(1.54–3.04)	<0.001
Distance to nearest health facility (km)								
		3.06	0.96	(0.87–1.06)	0.410	-	-	-
Population density (100 people/km^2^)								
		1.90	1.11	(0.99–1.24)	0.069	-	-	-
Source of information								
	Radio							
	No	10	REF	-	-	REF	-	-
	Yes	21	2.46	(1.83–3.34)	<0.001	3.04	(2.20–4.21)	<0.001
	Tv							
	No	18	REF	-	-	REF	-	-
	Yes	21	1.31	(0.98–1.74)	0.065	1.68	(1.20–2.33)	<0.001
	Health centre							
	No	19	REF	-	-	REF	-	-
	Yes	16	0.79	(0.62–1.00)	0.050	1.27	(0.96–1.69)	0.094
	Village leaders							
	No	20	REF	-	-	REF	-	-
	Yes	14	0.77	(0.60–0.97)	0.027	0.77	(0.59–1.02)	0.067
	Religious leaders							
	No	22	REF	-	-	REF	-	-
	Yes	15	0.66	(0.54–0.81)	<0.001	0.78	(0.61–0.99)	0.040
	Word of Mouth							
	No	22	REF	-	-	REF	-	-
	Yes	18	0.83	(0.65–1.06)	0.130	0.78	(0.59–1.03)	0.040
	Other							
	No	19	REF	-	-	-	-	-
	Yes	14	1.05	(0.71–1.52)	0.800	-	-	-

*Full models adjusted for intervention arm, survey number, and cluster number

**Abbreviations:** SES: Socioeconomic status, AOR: Adjusted Odds Ratio, OR: Odds Ratio

The results of the Getis-Ord-Gi* test showed hotspots of households that did not avoid health centres and those that did avoid health centres because of the pandemic which were overlapped by the locations of statistically significant clusters detected using Kulldorf spatial scan statistic in SaTScan (Fig 2B). The two tests detected similar patterns with significant clusters of households that avoided health centres as a result of the pandemic in Covè centre (Cluster 4: RR = 3.17) and Ouinhi North (Cluster 10: RR = 3.91), and clusters of households that did not avoid health centres in Covè North (Cluster 1: RR = 0.12), Zagnanado/Covè (Cluster 5: RR = 0.27) and Ouinhi South (Cluster 11: RR = 0 and Cluster 12: RR = 0.16).

## Discussion

In this mixed-methods study, the quantitative and qualitative results together create a better understanding of the community-level impacts of the coronavirus pandemic on malaria prevention and health seeking behaviours in rural Benin. Although there were minimal observed impacts, our findings highlight the need for and the importance of efforts to sustain malaria prevention and control interventions during health emergencies. To understand the level of understanding of COVID-19 among the study participants, which is reflective of community-level knowledge, a knowledge score was calculated based on questions surrounding symptoms, modes of transmission, and preventative measures of the disease. The quantitative results show that a tenth of the participants had a good knowledge of COVID-19, while the FGDs provided further insight on this metric. Particularly, FGD participants had a varying understanding of the symptoms and modes of transmission of COVID-19, but they could identify and practice effective preventative measures such as social distancing, mask-wearing, and handwashing, coined as the “gestes barrière”. For example, participants wore masks to prevent inhaling SARS-CoV-2 infected dust, or participants socially distanced from one another to prevent touching an infected person’s sweat. Two metrics were used to assess the impact of the pandemic on malaria prevention, including LLIN usage and access. The quantitative results indicate that LLIN usage and access did not decrease as a result of the pandemic, but the qualitative results provide evidence that there were challenges in sharing LLINs during the pandemic due to social distancing measures which resulted in a shortage of LLINs. Lastly, changes in health seeking behaviours during the pandemic were assessed with a metric that asked participants whether they avoided health centres for urgent or routine visits during the pandemic. The quantitative results indicate that a fifth of the participants avoided health centres during the pandemic, but the qualitative results demonstrate that some participants went to health centres more often, less often or did not change their behaviours during the pandemic–all of which were not captured in the quantitative results.

Our results also indicate that accessible and audible sources of information, specifically radios, televisions, and public criers, are effective measures to disseminate novel and salient information during a health emergency. The qualitative findings highlight that radios broadcasted in a dominant language and public criers reached all the population in rural areas; including those who are illiterate, from any socioeconomic status, and without any time constraints. The quantitative and qualitative findings also point to the importance of providing information on preventative measures rather than focusing on educational campaigns on the novel disease (i.e., knowledge of symptoms and modes of transmission).

There are, however, negative effects of disseminating information during health emergencies that should be addressed. COVID-19 knowledge was a strong indicator of health centre avoidance because of the pandemic. This is likely due to early messaging during the pandemic which advised sick people to stay home to reduce the transmission of COVID-19. Those who were well informed were hesitant to visit health centres, even after stay-at-home orders were lifted. This association was also evident spatially, where there were clusters of people that avoided health care centres, but that also had good COVID-19 knowledge. This could have strong negative implications when it comes to disease management and transmission, when certain populations do not seek medical care when sick (i.e., testing and prompt treatment). Another misinterpretation of health information is evidenced by people social distancing while sleeping and no longer sharing LLINs. Although public health messaging urges people to social distance, it should not be at the expense of other significant infectious diseases causing mortality, like malaria.

Understanding the different population sub-groups is also crucial in tailoring messages or strategies during health emergencies. For instance, Ouinhi had the greatest population of people from the Holli ethnic background compared to Covè and Zagnanado; and had significant clusters of people with lower levels of COVID-19 knowledge. Those from the Holli ethnic background were described as being more isolated from people outside their ethnic group, which poses a challenge during health emergencies–including the current COVID-19 vaccination campaign in the district (Dr. P. Davodoun, personal communications). Therefore, typical approaches and sources of information may not be as effective for people in Ouinhi compared to other districts.

This study also has some limitations that should be considered. In this study, LLIN usage and access did not decrease because of the pandemic. However, this study leveraged an ongoing LLIN trial which began in 2019, and the country had a national LLIN campaign in 2020. In fact, Benin was one of 31 malaria endemic countries scheduled to have a national LLIN distribution in 2020 [2]. In response to the pandemic, the LLIN distribution in Benin was modified and delayed by a couple of weeks. Up until 2020, households in Benin received vouchers that they had to bring to a centralized distribution site to receive their LLIN. For the 2020 distribution, community health workers went door-to-door to distribute the LLIN [29, 30]. This has major implications for the impact of LLIN usage and access during the pandemic since LLINs were not only distributed with the trial, but houses received LLINs through the national campaign. However, this highlights the importance of maintaining malaria control during health emergencies since only three-quarters of planned LLIN distributions in malaria endemic countries in 2020 had been completed by the end of their planned distribution year [2]. Indeed, if Benin did not have a LLIN campaign and their LLIN distribution reduced by 25% (described as scenario 1 in the modeling scenarios outlined by the WHO Global Malaria Programme), the country could have seen a 22% increase in malaria cases and death [31].

Another limitation was the method in which the additional KAP questions were administered during the cross-sectional study. All the possible answers for each domain (i.e., all the possible symptoms of COVID-19) were listed and asked to the participant. This approach led to the generation of a KAP score that reflects a participant that answered at least 80% of the questions correctly, which reflects the low knowledge score found in this study. With spontaneous responses (i.e., the respondent volunteers a response), a different knowledge score could have been calculated with a binary variable for good COVID-19 knowledge reflecting a participant naming at least one correct answer per domain (i.e., naming at least one COVID-19 symptom rather than identifying at least 80% of the symptoms correctly)–which might better reflect the responses from the FGDs [32, 33]. Although a minority of respondents had a good knowledge of COVID-19, the metric that was calculated in this study pointed to important statistical associations with health seeking behaviours and spatial clustering of households with good and poor COVID-19 knowledge in the study area.

We also did not account for transportation challenges and service disruptions in evaluating factors associated with health centre avoidance because of the pandemic. Transportation challenges for health care workers and patients as a result of the pandemic (i.e., financial limitations, social distancing measures, price of fuel) have all been noted as barriers in accessing health care centres, while malaria service disruptions have been noted in Rwanda, Uganda, Nigeria and Ghana [34–39]. Despite this limitation, the mixed-methods study design allowed us to reveal differences in health-seeking behaviour within our study participants. Prior to the pandemic, there was a dichotomy of people avoiding and going to health centres when they were sick. Consistent with other studies, the pandemic exacerbated the dichotomy of people going to and avoiding health centres [36, 38, 40]. Namely, fear of exposure to COVID-19 at the hospitals and financial barriers associated with health centres (i.e., cost of care, reduced income) led some people to avoid health centres because of the pandemic, while fear of COVID-19 and its overlapping symptoms with malaria lead some people to go to the health centre more often than before the pandemic.

## Conclusion

This study assessed community-level impacts of the COVID-19 pandemic on malaria prevention and health-seeking behaviours in rural Benin. This study shows the importance of sustaining malaria prevention and control and highlights recommendations for disseminating important information during health emergencies. These results can be used to provide insight and tailor malaria prevention and control for future health emergencies.

## Supporting information

S1 Table Knowledge of COVID-19 and malaria of the study participants by districts (n = 3858). (DOCX)
