# Supplementary material for: Community-level impacts of the coronavirus pandemic on malaria prevention and health-seeking behaviours in rural Benin: A mixed methods study
Source: PLOS Glob Public Health. 2023 May 19;3(5):e0001881. doi: 10.1371/journal.pgph.0001881 (PMC10198503; doi:10.1371/journal.pgph.0001881)
Supplement: S1 Table — (DOCX) [file pgph.0001881.s001.docx]

**S1 Table:** Knowledge of COVID-19 and malaria of the study participants by districts (n = 3858)

|  |  | Total  (n = 3858) | Cove  (n = 505) | Ouinhi  (n = 1242) | Zagnanado  (n = 2111) |
| --- | --- | --- | --- | --- | --- |
| COVID-19 Transmission | |  |  |  |  |
|  | Cough | 2916 (75.58) | 371 (73.47) | 923 (74.32) | 1622 (76.84) |
|  | Infected Surface | 1858 (48.16) | 304 (60.20) | 542 (43.64) | 1012 (47.94) |
|  | Contaminated meat/dairy | 1468 (38.05) | 208 (41.19) | 441 (35.51) | 819 (38.80) |
|  | Infected individual | 2216 (57.44) | 318 (62.97) | 718 (57.81) | 1180 (55.90) |
| COVID-19 Symptoms | |  |  |  |  |
|  | Fever | 2818 (73.04) | 362 (71.68) | 867 (69.81) | 1589 (75.27) |
|  | Cough | 2973 (77.06) | 378 (74.85) | 965 (77.70) | 1630 (77.21) |
|  | Shortness of breath | 1248 (32.35) | 166 (32.87) | 438 (35.27) | 644 (30.51) |
|  | Sore throat | 680 (31.94) | 72 (26.67) | 258 (37.34) | 350 (29.97) |
|  | Runny nose | 1361 (35.28) | 221 (43.76) | 378 (30.43) | 762 (36.10) |
|  | Muscle aches | 862 (22.34) | 131 (25.94) | 285 (22.95) | 446 (21.13) |
|  | Headache | 1709 (44.30) | 230 (45.54) | 478 (38.49) | 1001 (47.42) |
|  | Fatigue | 1647 (42.69) | 212 (41.98) | 508 (40.90) | 927 (43.91) |
|  | Diarrhea | 954 (24.73) | 133 (26.34) | 258 (20.77) | 563 (26.67) |
|  | Loss of taste | 707 (18.33) | 151 (29.90) | 168 (13.53) | 388 (18.38) |
| COVID-19 Protection | |  |  |  |  |
|  | Handwashing | 3549 (91.99) | 453 (89.70) | 1165 (93.80) | 1931 (91.47) |
|  | Touching eyes/nose/mouth | 1256 (32.56) | 166 (32.87) | 350 (28.18) | 740 (35.05) |
|  | Using disinfectants | 994 (25.76) | 136 (26.93) | 257 (20.69) | 601 (28.47) |
|  | Stay home when sick | 922 (23.90) | 171 (33.86) | 200 (16.10) | 551(26.10) |
|  | Using herbal supplements | 2843 (73.69) | 359 (71.09) | 930 (74.88) | 1554 (73.61) |
|  | Cover mouth/nose when cough/sneeze | 2379 (61.66) | 324 (64.16) | 698 (56.20) | 1357 (64.28) |
|  | Facemask | 3192 (82.74) | 384 (76.04) | 948 (76.33) | 1860 (88.11) |
|  | Social distancing | 2292 (59.41) | 275 (54.46) | 758 (61.03) | 1259 (59.64) |
|  | Traditional treatments | 2674 (69.31) | 336 (66.53) | 870 (70.05) | 1468 (69.54) |
|  | Public gathering | 2360 (61.17) | 280 (55.45) | 777 (62.56) | 1303 (61.72) |
|  | Going to health clinic for visit that could be postponed | 624 (16.17) | 74 (14.65) | 210 (16.91) | 340 (16.11) |
|  | Going to health clinic when you have a fever | 569 (14.75) | 60 (11.88) | 204 (16.43) | 305 (14.45) |
|  | Visiting family/friends | 683 (17.70) | 52 (10.30) | 217 (17.47) | 414 (19.61) |
|  | Buying drugs that treat COVID-19 | 2636 (68.33) | 365 (72.28) | 841 (67.71) | 1430 (67.74) |
|  | Buying PEE | 2605 (67.52) | 319 (63.17) | 982 (79.07) | 1304 (61.77) |
| Malaria Transmission* | |  |  |  |  |
|  | Mosquitoes | 2072 (97.32) | 263 (97.41) | 678 (98.12) | 1131 (96.83) |
|  | Water | 1827 (85.81) | 241 (89.26) | 602 (87.12) | 984 (84.25) |
|  | Food | 1829 (85.91) | 244 (90.37) | 601 (86.98) | 984 (84.25) |
|  | Air | 1883 (88.45) | 244 (90.37) | 613 (88.71) | 1026 (87.84) |
|  | Animal | 1709 (80.27) | 244 (90.37) | 548 (79.31) | 917 (78.51) |
|  | Season | 1295 (60.83) | 166 (61.48) | 402 (58.18) | 727 (62.24) |
|  | Sunshine | 1231 (57.82) | 172 (63.70) | 362 (52.39) | 697 (59.67) |
|  | Witchcraft | 1500 (70.46) | 186 (68.89) | 470 (68.02) | 844 (72.26) |
|  | Person-to-person | 1568 (73.65) | 198 (73.33) | 492 (71.20) | 878 (75.17) |
| Malaria Symptoms* | |  |  |  |  |
|  | Fever | 2105 (98.87) | 267 (98.89) | 688 (99.57) | 1150 (98.46) |
|  | Fatigue | 1927 (90.51) | 247 (91.48) | 623 (90.16) | 1057 (90.50) |
|  | Headache | 1917 (90.04) | 235 (87.04) | 636 (92.04) | 1046 (89.55) |
|  | Diarrhea | 701 (32.93) | 51 (18.89) | 261 (37.77) | 389 (33.30) |
|  | Nausea | 1273 (59.79) | 117 (43.33) | 445 (64.40) | 711 (60.87) |
|  | Stomach-ache | 652 (30.62) | 70 (25.93) | 267 (38.64) | 315 (26.97) |
|  | Convulsion | 665 (31.24) | 63 (23.33) | 283 (40.96) | 319 (27.31) |
| Data are displayed as n (%)  *Denominator for knowledge of transmission was n=2129 | | | | | |
